# Systems‐theoretic process analysis of a CT‐guided online adaptive radiation therapy system in a multi‐vendor environment

**DOI:** 10.1002/acm2.70330

**Published:** 2025-12-10

**Authors:** Colleen Foote, David M McClatchy, Susu Yan, Sven Olberg, Kyla Remillard, Nathaniel Miles, Jennifer Pursley

**Affiliations:** ^1^ Division of Radiation Biophysics Department of Radiation Oncology Massachusetts General Hospital Boston Massachusetts USA; ^2^ Harvard Medical School Boston Massachusetts USA

**Keywords:** adaptive therapy, hazard analysis, safety

## Abstract

**Background:**

Online adaptive radiation therapy (ART) is a relatively new process, and it is recommended that institutions starting an online ART program conduct a risk analysis to identify potential hazards. While Failure Modes and Effects Analysis (FMEA) is common, Systems‐Theoretic Process Analysis (STPA) has also been used to evaluate online ART workflows.

**Purpose:**

An STPA hazard analysis was performed for a CT‐guided online ART system in a multi‐vendor environment. The goal was to identify potential risks and mitigations to guide the development of adaptive workflows and the quality management (QM) program.

**Methods:**

The STPA hazard analysis was performed in four steps. First, process maps for online ART were generated to describe the interactions between users and systems. In the second step, the process maps were refined to a single control structure diagram model. In the third step, potential unsafe control actions (UCAs) were enumerated by the physicists involved in the analysis. Finally, mitigation strategies to address the UCAs were identified.

**Results:**

A total of 496 UCAs were identified for 119 control actions, of which 239 (48.2%) were prioritized for mitigation due to having low or medium levels of detectability. The most frequent causal scenarios were accidental omission (20.1%), rushing (17.2%), and lack of training (15.9%). The most common consequences were delays (26.8%) and having to repeat work (13.5%). The two mitigation strategies considered to address the most causal scenarios were requiring trained adaptive staff (28.9%) and having physics oversight (19.9%).

**Conclusions:**

The STPA led to valuable insights into the potential causes of unsafe control actions and various mitigation strategies that were used to develop the QM program. Notably, most UCAs were attributable to interactions between users and the system, rather than system failures. It is recommended that every institution starting an online ART program perform a risk assessment for their environment.

## 1 INTRODUCTION

The American Association of Physicists in Medicine (AAPM) report of Task Group 100 (TG‐100) highlighted that errors in radiation therapy were more often the result of failures in workflow and process than failures related to equipment and software.^1^ Online adaptive radiation therapy (ART) is a relatively new process where a new radiation treatment plan is generated and delivered based on an image acquired during the treatment session. When new technologies are introduced into the clinic, the quality management (QM) program must be evaluated and updated accordingly,^2^ and the technology and workflows for online ART differ substantially from conventional radiation therapy workflows.^3^, ^4^ It is recommended that institutions implementing an online ART program conduct a hazard analysis on adaptive procedures.^5^

Failure Modes and Effects Analysis (FMEA) has been used to perform prospective risk analysis for commercial online ART systems, primarily for MR‐linacs.^3^, ^6^, ^7^, ^8^, ^9^, ^10^ FMEA is performed by having a multidisciplinary team create a process map which identifies each step of a workflow. For each step in the process map, the team attempts to predict any potential failure mode that could occur during that step. To quantify the risk, each failure mode is assigned three numerical scores predicting the likelihood of occurrence, how easily detectable the failure is, and the severity of the failure if it were not detected. FMEA is discussed in detail with examples in the report of AAPM Task Group 100.^1^

Systems‐Theoretic Process Analysis (STPA) is a risk analysis method that focuses on the interplay of software systems with users and considers that errors may happen due to both component failure and unsafe interactions of the system components.^11^ STPA is performed by first creating a description of the system being analyzed, including all organizational and system components. The next step is to create a hierarchical control structure which details control actions and the feedback from these actions. Then unsafe control actions, which can result in a hazardous situation, are identified and potential causes are determined. A comparison of FMEA and STPA for radiation oncology was performed in Pawlicki et al.,^12^ which determined that while FMEA and STPA both ended up with causal scenarios, the two approaches should not be expected to give the same results, and STPA facilitates a hazard analysis for a *de novo* system where the failure modes are not necessarily known in advance. STPA has been used for prospective hazard analysis in radiation therapy for the Halcyon system^13^ and with mental decision models for the Ethos adaptive system.^14^

Several previous studies have performed hazard analyses for a CT‐guided online adaptive system, the Ethos (Varian Medical Systems, Palo Alto, CA) in an environment with ARIA (Varian Medical Systems, Palo Alto, CA) as the oncology information system.^9^, ^10^, ^14^, ^15^, ^16^, ^17^ Although the results of each study differ, there are common themes such as the importance of multidisciplinary staff coordination,^9^, ^14^ the existence of failures driven by the system's automated processes,^15^, ^17^ and the benefit of checklists to mitigate failures.^9^, ^16^ Ethos can also be installed in an environment with MOSAIQ (Elekta AB, Stockholm, Sweden) as the oncology information system, although we are not aware of any published hazard analysis results for this configuration. In 2022, our institution installed Ethos in a MOSAIQ environment, and we decided to perform an STPA hazard analysis for this configuration.

This study reports on an STPA hazard analysis performed at a single institution starting an Ethos ART program in a multi‐vendor environment with MOSAIQ as the oncology information system. The aim of this study was to identify hazards for a CT‐guided online adaptive platform and use this information to guide the development of adaptive workflows, staff training, and the QM program.

## 2 METHODS

The hazard analysis was performed by applying STPA with four main steps: (1) defining the scope of the analysis, (2) modeling the control structures, (3) identifying unsafe control actions and considering the causal scenarios and consequences, and (4) designing mitigation strategies. The team performing the STPA consisted of four physicists, one physics resident, and two dosimetrists who all received Varian training for Ethos and participated in the Ethos adaptive workflows. Radiation oncologists and therapists who participated in Ethos ART were also asked for feedback on the workflows and safety concerns. At the time this study began, treatments on Ethos at this institution had not yet started and no team members had prior experience with Ethos ART. The study continued through the first year of treatments on the Ethos, and the experience gained during that time contributed to the analysis.

For the first step, defining the scope of the analysis, process maps were generated in Lucidchart (Lucid Software Inc., USA) for each major step in the treatment delivery process (CT simulation, treatment planning and approval, approved plan preparation, pretreatment QA, and treatment delivery) for adaptive treatments on Ethos in this clinic's multi‐vendor environment. The maps summarized the interactions between systems and humans showing the flow of data and control actions.

**FIGURE 1:**
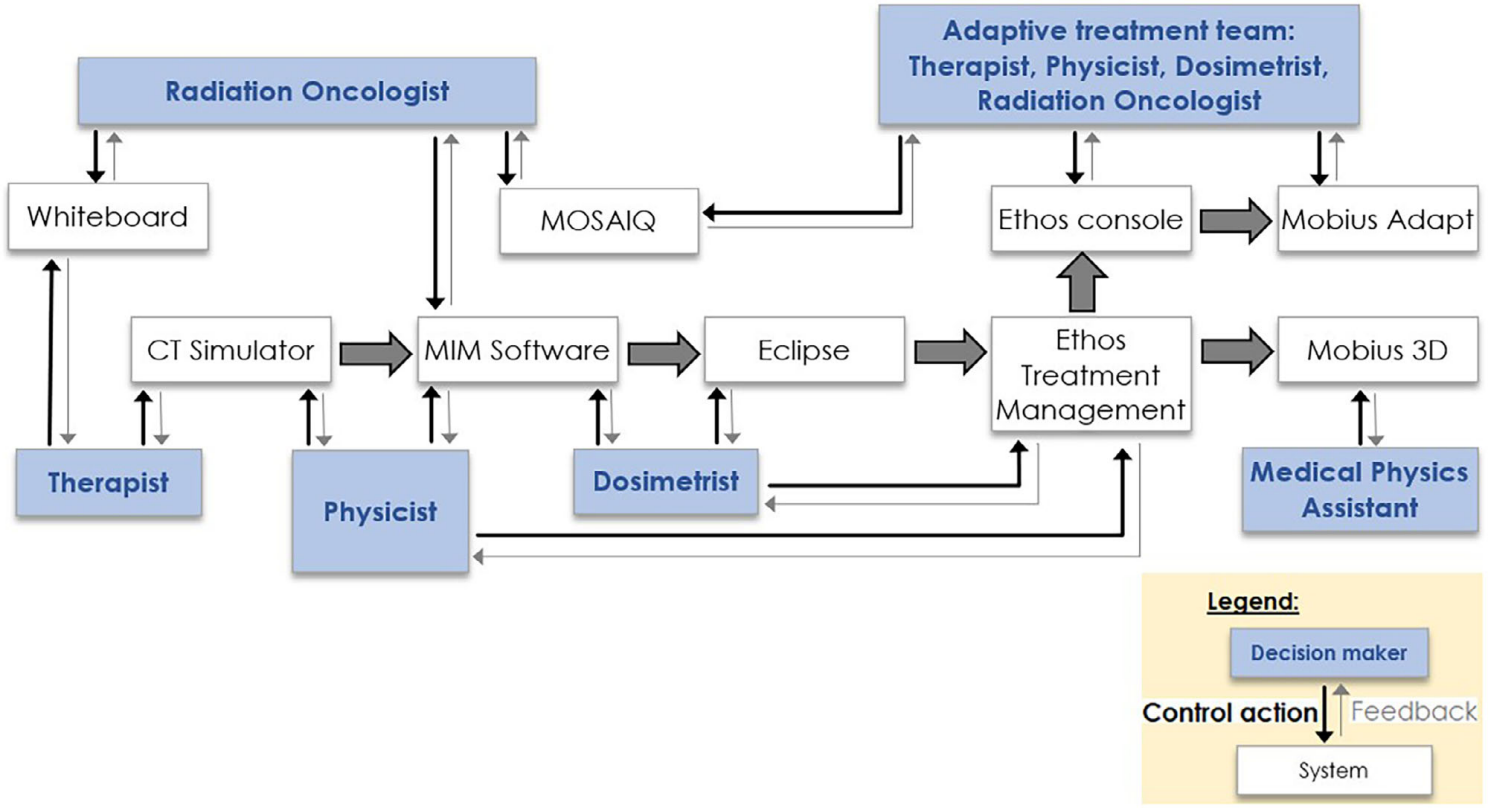
Simplified diagram of the control structure for Ethos online adaptive therapy investigated in this hazard analysis. Blue rectangles indicate staff decision makers and white rectangles indicate software systems. Large gray arrows show the flow of treatment planning data between the systems. Black arrows indicate control actions from staff to systems and gray arrows indicate feedback from the system to staff. A total of 119 control actions were identified in this workflow.

To model the control structure, the process maps for adaptive treatments were refined to a single control structure diagram model of the system. A simplified diagram of the control structure used for this analysis is shown in Figure 1. The control structure contained both control actions and control loops, which provided feedback to the controllers after each control action. Staff participating in the adaptive process served as the controllers. The controllers included radiation oncologists, therapists, dosimetrists, medical physics assistants, or physics residents, and physicists. The first system in the workflow was the clinic's in‐house Whiteboard system,^18^ which was used to place an order for CT simulation for adaptive therapy. After the simulation, the radiation oncologist contoured the scan in MIM (MIM Software Inc, Cleveland, OH) and added a prescription for adaptive therapy to MOSAIQ. The dosimetrist transferred the CT scan and contours from MIM to Eclipse (Varian Medical Systems, Palo Alto, CA) for import into Ethos Treatment Management (ETM). After the adaptive plan was developed in ETM, it was exported to Mobius 3D (Varian Medical Systems, Palo Alto, CA) for a secondary dose calculation and logfile QA, which was performed by Medical Physics Assistants. A physicist performed a pretreatment plan check on the plan in ETM. During adaptive treatment, the adaptive treatment team, composed of two therapists, a physicist, dosimetrist, and radiation oncologist, interacted with the Ethos console to contour and plan on the daily image. Prior to delivery, the adapted plan was sent to Mobius Adapt, an online version of Mobius 3D, for secondary dose calculation. The individual control actions in this adaptive workflow were converted to an Excel spreadsheet to facilitate the next step, identifying unsafe control actions.

Unsafe control actions (UCAs) were identified by asking the following questions at each workflow step: what happens when the control action is (1) not provided, (2) provided incorrectly, (3) provided at the wrong time or in the wrong order, or (4) provided too long or stopped too soon. The physicists brainstormed the answers to these questions for several of the workflow actions together as a team. After answering these questions for a particular workflow step, the team discussed the resulting potential accidents and hazards. This analysis used the five system‐level accidents and hazards described in Pawlicki et al.^14^ which are specific to radiation oncology, shown in Table 1. Next, the team listed possible causal scenarios that could lead to each UCA and then listed the potential consequences if the UCA were not caught. It was noted that many causal scenarios and consequences were repeated across multiple workflow steps. Twelve causal scenarios and ten consequences which repeated more than 10 times were assigned a code, shown in Table 2, which facilitated quantitative analysis. After completing the analysis for several steps together, the remaining steps were divided among the four physicists to complete independently, with a review of each workflow step performed by a second physicist and physics resident. The hazard analysis team had an hourly meeting once per month for a year and each team member spent several hours working on the analysis between meetings.

**TABLE 1:** System‐level accidents and associated hazards, denoted with the initials A and H, respectively.These were defined for radiation oncology treatments in Pawlicki et al.^13^

System‐level accidents		System‐level hazards	
A1	Patient injured from radiation	H1	Radiation delivered incorrectly
A2	Staff injured from radiation	H2	Staff exposed to radiation
A3	Injury during treatment not from radiation	H3	Possibility of non‐radiation injury
A4	Damage to equipment	H4	Equipment subject to stress
A5	Damage to satisfaction or hospital reputation	H5	Workflow subject to stress or delays

**TABLE 2:** Twelve causal scenarios, 10 consequences, and seven mitigation strategies, denoted with the initials S, C, and M, respectively, were identified during the analysis of the unsafe control actions (UCAs) for this adaptive workflow.

Causal scenarios		Consequences		Mitigation strategies	
S1	Distraction or accidental omission	C1	Delay or loss of time	M1	Requiring trained adaptive staff
S2	Rushing or feeling rushed	C2	Repeating work	M2	Physics oversight or peer review
S3	Lack of or improper training	C3	Insufficient documentation	M3	Pre‐treatment team huddle
S4	Lack of experience	C4	Incorrect dose to patient	M4	Multiple staff review
S5	Complacency	C5	Staff confusion or loss of morale	M5	Documentation
S6	Communication breakdown	C6	Incorrect plan used for treatment	M6	Add to timeout or QA procedure
S7	Software failure or downtime	C7	Excess dose to patient	M7	Automation
S8	Intentional violation	C8	Non‐radiation discomfort for patient		
S9	Manual entry error	C9	Incorrect contours		
S10	Confusion between multiple cases	C10	Incorrect scan acquired		
S11	Not physically present				
S12	Waiting for instructions				

The last step of the STPA was to design mitigation strategies for the identified hazards. After completing the review of all workflow steps, a detectability screening was applied to prioritize the hazardous scenarios for mitigation. The detectability screening utilized three levels: (1) high for a scenario that would be detectable with current QA procedures, (2) medium for a scenario that may be detectable with current QA procedures but which those QA procedures were not specifically designed to detect, and (3) low for a scenario that was unlikely to be detected with current QA procedures. Any scenarios that could result in A1/H1 events (patient injured from radiation/radiation delivered incorrectly as shown in Table 1) and were scored at low or medium levels of detectability were included for discussion. The physicists and physics resident discussed possible mitigation strategies to address these scenarios. Two dosimetrists who participated in Ethos adaptive treatments then reviewed the full analysis and provided suggestions that were incorporated into the mitigation strategies. Radiation oncologists and therapists reviewed the workflows and provided feedback and recommendations. Because the study continued during the first year of adaptive treatments, experience with Ethos ART gained by the multidisciplinary team during that time was incorporated into the analysis and the institution's adaptive QM program.

## 3 RESULTS

A total of 119 control actions were identified in the workflow spreadsheet based on the control diagram shown in Figure 1. A total of 496 unsafe control actions were identified for the online ART workflow, an average of 4 per workflow step. According to the definitions in Table 1, 262 (35.8%) were categorized as H1/A1, 0 (0%) as H2/A2, 19 (2.6%) as H3/A3, 2 (0.3%) as H4/A4, and 448 (61.3%) as H5/A5. Many UCAs were identified related to information transfer between multiple systems and manual actions required due to the multi‐vendor environment. Most of these UCAs occurred in the workflow either before or after the online adaptive process, such as during the CT simulation, during treatment planning, or when recording the adaptive treatment in MOSAIQ. The adaptive treatment happens on the Ethos console itself so there is minimal interaction with other systems; the UCAs that could occur during adaptive treatment were primarily caused by users interacting with the Ethos software. Each UCA was considered for causal scenarios and consequences.

**FIGURE 2:**
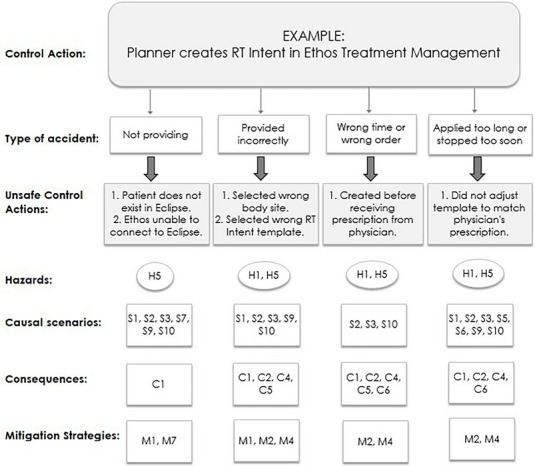
Example of the STPA analysis for one control action, a planner creating the RT Intent in Ethos Treatment Management, which is the first step in generating a treatment plan on the CT simulation image. The Hazards (H1–H5) are defined in Table 1 and the Causal scenarios (S1–S12), Consequences (C1–C10), and Mitigation strategies (M1–M7) are defined in Table 2.

**FIGURE 3:**
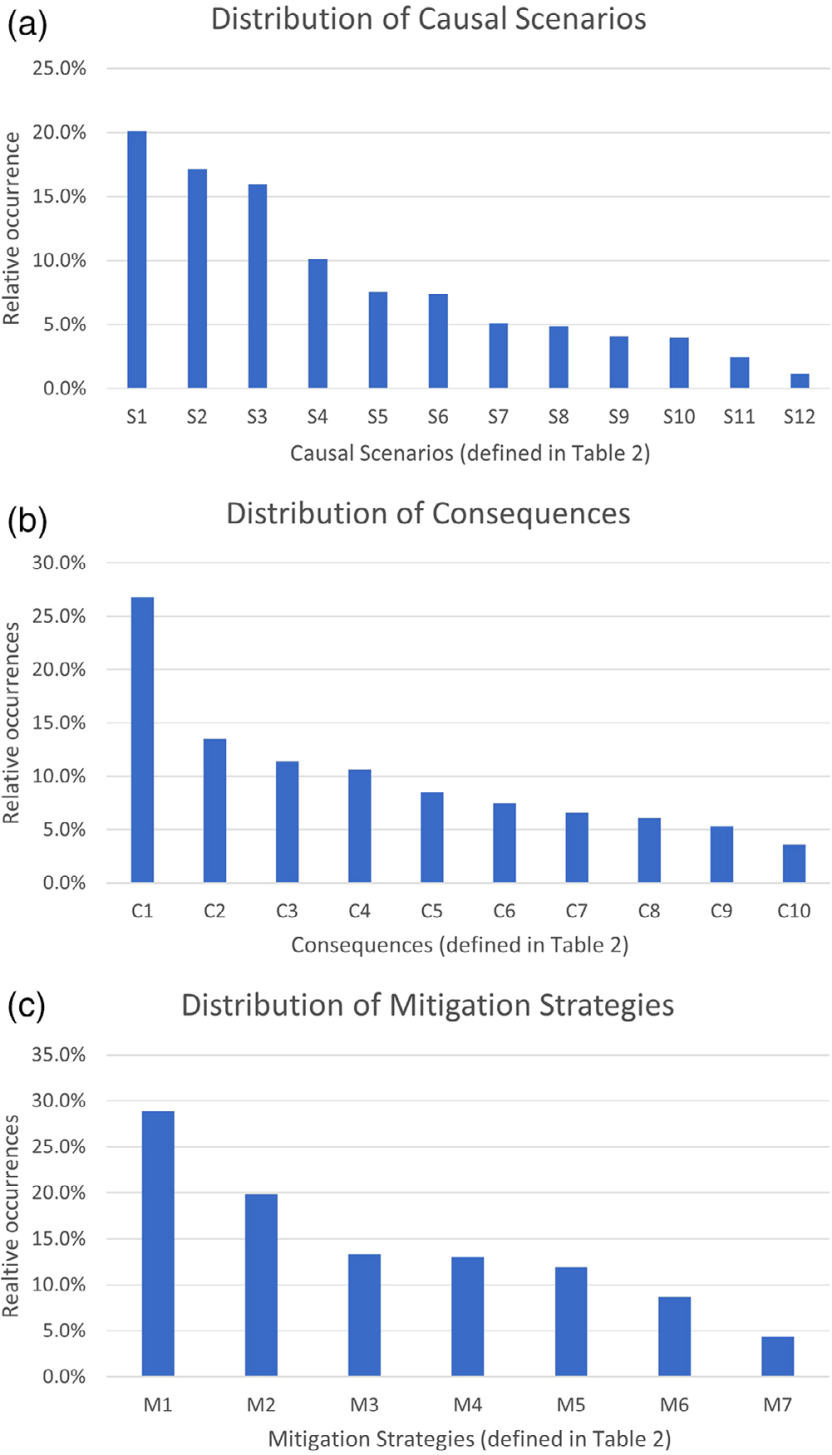
Causal scenarios (S1–S12), consequences (C1–C10), and mitigation strategies (M1–M7) are defined in Table 2. (a). Distribution of 2026 causal scenarios identified during the hazard analysis. (b). Distribution of 1249 consequences identified during the hazard analysis. (c). Distribution of 277 mitigation strategies identified to address the 239 unsafe control actions with low or medium detectability scores.

Multiple causal scenarios could contribute to a single UCA, and 2026 instances of the 12 repeated causal scenarios were identified for the 496 UCAs. Each UCA could also lead to more than one possible consequence, and 1249 instances of the ten repeated consequences were identified for the 496 UCAs. An example of the STPA analysis for one control action, that of the planner creating the RT Intent prescription in the Ethos Treatment Management system, is shown in Figure 2. Distributions of the causal scenario and consequence occurrences are shown in Figure 3a,b.

The detectability screening of the 496 UCAs resulted in 257 (51.8%) categorized as high detectability, or detectable with current QA procedures. These UCAs were not considered for mitigation strategies. All remaining UCAs were considered for mitigation strategies; of these, 144 (29%) were categorized as medium detectability, and 95 (19.2%) were categorized as low detectability. Thus, a total of 239 UCAs were prioritized for mitigation discussions. Many of the low‐detectability UCAs were related to the controller not reviewing information, such as the patient setup information not being transferred from MOSAIQ to Ethos because the therapist forgot or did not know where to enter setup information in Ethos. Contouring on the daily image resulted in many UCAs considered medium detectability because contours were reviewed by a radiation oncologist prior to adaptive plan generation. Repeated mitigations fell into seven categories, shown in Table 2, and the distribution of their use for UCAs is shown in Figure 3c.

The results of the STPA hazard analysis showed several trends in unsafe control actions that could occur during online adaptive therapy. The three most frequent causal scenarios of UCAs were distraction or accidental omission (20.1%), rushing or feeling rushed (17.2%), and lack of or improper training (15.9%). The most common consequences of UCAs were a delay or loss of time (26.8%) and having to repeat work (13.5%) which could also result in delays. This agreed with the finding that the most common associated hazard in this analysis was having the workflow subjected to stress or delays (61.3%). For online adaptive therapy, delays could negatively impact the treatment by allowing the anatomy to change such that the new treatment plan was no longer appropriate. The two mitigation strategies that addressed the most causal scenarios were requiring trained adaptive staff (28.9%) and having physics oversight or another form of peer review (19.9%).

## 4 DISCUSSION

Based on the results of this analysis, several new quality management steps were implemented. Those steps included designing credentialing programs for all adaptive staff; having trained adaptive physicists review adaptive plans prior to physician review; restricting adaptive plan checks to trained adaptive physicists; having the physicist lead a team huddle prior to each adaptive treatment; having the physicist write a posttreatment note in MOSIAQ to summarize any considerations for the next treatment team; and by having another adaptive physicist perform a posttreatment peer review after every adaptive fraction. While these QM steps have not prevented UCAs from occurring, they have helped to minimize their impact by raising staff's awareness of vulnerabilities. It should be noted that these mitigations were determined to be important in an environment in which online ART is new and relatively rare; if online ART becomes routine at an institution, some of these mitigations may become unnecessary. Notably, automation was the least frequently utilized mitigation strategy in this analysis. The Ethos online adaptive workflow is already highly automated, and many UCAs resulted from a lack of human review as also seen in previous Ethos hazard analyses.^16^, ^17^ However, an automated QA tool could identify errors in contours or planning that might otherwise be missed, as found in a study by Rippke et al.^19^ which assessed the benefit of an automated tool for an adaptive MR‐linac workflow.

Most previous hazard analyses for online adaptive workflows used FMEA and were for an MR‐guided workflow rather than CT‐guided. Noel et al. performed an FMEA for adaptive IMRT and concluded it was more vulnerable to failures in the segmentation and treatment planning process than non‐adaptive IMRT.^6^ Kluter et al. performed an FMEA for adaptive MR‐guided treatments and decided that the primary mitigations were to use standardized workflows, clearly defined protocols, and checklists.^7^ Nishioka et al. also performed an FMEA for adaptive MR‐guided treatments and found the most hazardous processes to be structure segmentation, treatment planning, and delivery.^8^ Wegener et al. performed an FMEA for Ethos online ART and gave a description and mitigation strategy for the 20 highest‐ranked events, many of which were related to not reviewing all plan parameters.^9^ After 1 year of adaptive treatments, they used all reported safety events to update their original FMEA analysis; they found some of their original concerns were not applicable and that there were several failures not anticipated by the original analysis.^15^ Wang et al. performed an FMEA for Ethos online ART, designed workflows and checklists which reduced the risk priority numbers of the top 20 failure modes, and then reported on the results.^10^ Rahman et al. performed an FMEA specifically for the Ethos adaptive planning process and developed a reference planning checklist to mitigate failure modes.^16^ Zheng et al. performed a retrospective analysis of over 1000 Ethos adaptive treatments to identify recurring failure modes, which could be classified in three domains as system‐driven, patient‐driven, or treatment planning, and execution failures.^17^ Wong et al. used STPA to study operational decision‐making during Ethos online ART and concluded that errors could be reduced by reducing the number of staff needed to perform a treatment and ensuring those staff were adequately trained.^12^ Similar to previous analyses, the results of the STPA hazard analysis presented here also found that many unsafe conditions could result from staff not reviewing all information or not being trained on the adaptive system.

Because this study was ongoing when Ethos online ART treatments started, updates to workflows and to the hazard analysis were made at the same time, and it was not possible to measure how effectively the mitigations reduced error rates. Despite this study limitation, adjusting the workflows in real time based on feedback and observations from members of the adaptive team was felt to be essential for quality management. Many of the QM elements implemented from the beginning were found to be helpful, such as writing detailed standard operating procedures and developing checklists for tasks with many manual steps such as plan writeup, physics plan check, and the online ART workflow. Most feedback centered on making small updates to the documentation or workflows based on experiences gained during treatment.

A limitation of this study, as with all prospective risk assessments, is the qualitative nature of the analysis. The list of UCAs, causal scenarios, and mitigations rely primarily on the judgement of the physicists leading the analysis. Efforts were made to address this concern by having multiple physicists review each step, having dosimetrists review the full study, and involving Radiation Oncologists and therapists in adaptive workflow QM. These steps cannot guarantee that a different team performing this analysis would find the same results, but it should improve the robustness of the analysis and its conclusions. Additionally, no UCAs which were not identified in this analysis have been observed during adaptive treatments, and to date the clinic has treated over 600 fractions of Ethos online ART.

Although this study may be useful to help guide other new adopters of Ethos adaptive technology, the interactions between staff and systems will vary by institution and with the exact systems in use. Therefore, each institution should perform its own hazard analysis and identify UCAs based on its systems and intended workflow. The mitigation strategies may be similar to the ones employed here, but the exact form in which they are implemented may be different.

## 5 CONCLUSION

A Systems Theoretic Process Analysis was used to evaluate an Ethos online adaptive workflow in a multi‐vendor environment and led to valuable insights into the potential causes of hazards. The mitigation strategies that addressed the most common causal scenarios were requiring trained adaptive staff and having physics oversight or another form of peer review, and these insights were used to develop a quality management program for the adaptive workflow. It is recommended that every institution starting an online ART program perform a risk assessment for their environment.

## AUTHOR CONTRIBUTIONS

All persons listed as authors contributed directly to the design or interpretation of the data for this work, and to the drafting, revision, and final approval of this manuscript.

## CONFLICT OF INTEREST STATEMENT

D.M.M. reports consulting for DoseOptics, LLC. S.Y. reports being an author of US patent 16/607664 on System and Method for Gantry‐less Particle Therapy.
